# Association of Dietary Behaviors and Sleep Quality: Results from the Adults Chronic Diseases and Risk Factors Survey of 2015 in Ningbo, China

**DOI:** 10.3390/ijerph15091823

**Published:** 2018-08-23

**Authors:** Fanqian Kong, Hui Li, Guodong Xu, Yanyan Ying, Qinghai Gong, Jinshun Zhao, Xiaohong Zhang, Lina Zhang, Shiwei Liu, Liyuan Han

**Affiliations:** 1 Department of Preventive Medicine, Zhejiang Provincial Key Laboratory of Pathophysiology, School of Medicine, Ningbo University, Ningbo 315211, China; 15058431101@163.com (F.K.); 18815281025@163.com (G.X.); zhaojinshun@nbu.edu.cn (J.Z.); zhangxiaohong@nbu.edu.cn (X.Z.); zhanglina@nbu.edu.cn (L.Z.); 2 Ningbo Municipal Center for Disease Control and Prevention, Ningbo 315010, China; lihui4329@163.com (H.L.); 15058438775@163.com (Y.Y.); gongqinghai@163.com (Q.G.); 3 National Center for Chronic and Noncommunicable Disease Control and Prevention, Chinese Center for Disease Control and Prevention, Beijing 100050, China

**Keywords:** dietary behavior, sleep quality, cross-sectional study, PSQI

## Abstract

*Objective*: We estimated the relationship between dietary behaviors and sleep quality in adults. *Methods*: Using data from the 2015 Ningbo Adult Chronic Diseases and Risk Factors Survey, a total of 5160 participants were included in this study. Sleep quality was measured by the Pittsburgh Sleep Quality Index (PSQI). PSQI score ≥ 7 was defined as poor sleep quality. Logistic regression was used to estimate the associations between dietary behaviors and sleep quality. Linear regression was used to explore the associations between dietary behaviors and PSQI total score. *Results*: Approximately 8.6% (*n* = 442) participants reported poor sleep quality. After adjusted for multivariates, there was 0.022 point increase in PSQI score for every gram increase in each meal consumption of soy sauce (*β* = 0.022, *p* = 0.001). Less intake of dark fruits, water and more intake of alcohol were associated with higher PSQI score (*β* = −0.394, *p* = 0.001; *β* = −0.246, *p* = 0.001; and *β* = 0.217, *p* = 0.005, respectively). *Conclusions*: About 8.6% (*n* = 442) adults reported poor sleep quality in Ningbo. Intake of soy sauce and alcohol were positively associated with poor sleep quality, and consumption of dark fruits and water were positively associated with good sleep quality.

## 1. Introduction

Sleep quality is very important for maintaining human beings’ health [1,2]. It is well known that poor sleep quality contributes significantly to cancer [3], depression [4], and dementia [5]. Unhealthy habits, such as smoking [6,7] and drinking [8,9,10,11] are associated with poor sleep quality. Besides, the sleep quality of adults has decreased along with lifestyle changes [12,13]. According to the Global Epidemiological Survey of Adult Insomnia, 28.0% of Chinese adults suffer from insomnia disorders [14].

Epidemiological evidence indicated a positive relationship between dietary behaviors and sleep quality [8,9,10,11,12,13,14,15,16]. Alcohol consumption was associated with a higher risk of sleep apnoea in adults [8]. Lower intake of fish and vegetables, higher intake of noodles and carbohydrates were associated with poor sleep quality among middle-aged female workers [15]. The timing of meals may also influence sleep quality; people with a nocturnal lifestyle, who consume most of their food at night, are particularly prone to suffer from low sleep duration [16]. To date, most studies were performed in Western countries, but there are huge differences in dietary behaviors between Western and Asian countries. For example, compared to the preference of fast food in Western countries, more edible oil and salt are added for the taste of the food in China [17]. To date, few studies have estimated the relationship between dietary behaviors and sleep quality in Chinese adults. Therefore, we aimed to investigate dietary behaviors and sleep quality in a representative sample of adults in Ningbo, China.

## 2. Methods

### 2.1. Study Participants

Ningbo, an economic center of Zhejiang Province, is one of the major coastal cities with a population of over 7 million, and a higher level of economic development than the general level in China (GDP per capita in 2014: 16, $112 vs $7065) [18,19]. The city is located near the East China sea with diverse and tasty seafood, which are commonplace on Ningbo families’ dining tables [20].

The 2015 Ningbo Adult Chronic Diseases and Risk Factors survey monitored the prevalence of chronic diseases and associated risk factors in a representative sample of adults in Ningbo. Participants aged between 17–74 and had lived in Ningbo for at least five years were included in the study. Multi-stage stratified cluster random sampling was applied. A total of 11 monitoring sites, which were comprehensively representative of the whole of Ningbo and comprising at least 630 included participants per monitoring site, were used for a final total of 5160 included participants in the study. The study was conducted by the Ningbo Center for Disease Control in 2015 and approved by the Ethics Committee of the Ningbo Municipal Center for Disease Prevention and Control (NO: 201702).

### 2.2. Demographic Variables

Demographic variables were collected by a validated questionnaire, which was designed by the Chinese Center for Disease Control and Prevention. This questionnaire is now widely used across the whole China. The Chinese Center for Disease Control and Prevention have invited a group of experts to improve and validate this questionnaire, however, no paper has been published on this yet. The demographic items included age, sex, body mass index (BMI), education levels (did not complete high school, high school graduate or some college, college graduate or more), occupation (unemployed, employed, student, retired), marital status (single, married, widowed, divorced), regular exercise (yes or no), monthly household incomes per capita (<$295, $295–370, $370–440, $440–515, >$515), smoking (yes or no), hypertension (yes or no), diabetes (yes or no), and dyslipidemia (yes or no). BMI was calculated as kg/m^2^ based on participants’ height and weight measured by a registered nurse during the baseline visit. Height was measured without shoes using a metal column height meter, and weight was measured without shoes and in light clothing to the nearest 0.1kg using an electronic weighing scale. Participants who have smoked at least 20 packs of cigarettes and have smoked for more than six months were defined as smokers in this study [21]. The level of systolic blood pressure (SBP) ≥ 140 mmHg and/or diastolic blood pressure (DBP) ≥ 90 mm Hg was defined as hypertension based on the Chinese Hypertension Treatment 2005 Guidelines [22]. The level of fasting blood glucose (FBG) levels ≥ 7.0 mmol/L and/or oral glucose tolerance test (OGTT)-2h glucose ≥ 11.1 mmol/L was defined as diabetes based on the Chinese Type 2 Diabetes Prevention and Control 2010 Guidelines [23]. In accordance with the Chinese Adults Dyslipidemia Prevention and Control 2007 Guidelines, the level of total cholesterol (TC) ≥ 6.22 mmol/L or high-density lipoprotein cholesterol (HDL-C) < 1.04 mmol/L or low-density lipoprotein cholesterol (LDL-C) ≥ 4.14 mmol/L was defined as dyslipidemia [24]. Blood pressure was measured by an electronic blood pressure meter. Before the measurement, the participants were requested to rest for 5–10 minutes. The blood pressure was measured at the right upper limb brachial artery blood pressure and in a seated posture. Each participant was needed to measure their blood pressure 3 times at 30-second intervals, and the average of the 3 measurements was the final blood pressure. Blood glucose was measured by the modified hexokinase enzymatic method. TC and HDL-C were measured enzymatically using commercial reagents, and LDL-C was calculated by the Fried Ewald equation [25].

### 2.3. Dietary Behaviors

Dietary behaviors were collected via a 16-item dietary questionnaire, which included condiments (salt, MSG, edible oil, and soy sauce), vegetables (dark vegetables and light vegetables, fruits (dark fruits and light fruits), water, tea, coffee and alcohol, eating out (eating breakfast out, eating lunch out, eating dinner out, and eating midnight snacks out). Different dietary behaviors were explored for the different durations. The dietary behaviors which were explored for the past week included the frequency of eating breakfast out, eating lunch out, eating dinner out, and eating midnight snack out. The dietary behaviors which were explored for the past month included the average consumption of salt, MSG (MSG is a kind of common condiment in China and is mainly used to increase the flavor of food), edible oil and soy sauce for each meal, and the daily frequency of eating dark vegetables (such as spinach, tomatoes, and purple cabbage), light vegetables (such as wax gourds and white radish), dark fruits (such as oranges, mangoes and kiwi fruit), and light fruits (such as apples, pears, and bananas). The dietary behaviors which were explored for the last year included the monthly intake of cups of tea and coffee, the monthly frequency of drinking alcohol, and the daily intake of cups (about 200 mL per cup) of water. The dietary behaviors questionnaire was designed and validated by the Chinese Center for Disease Control and Prevention. This questionnaire is widely used across the whole China. The Chinese Center for Disease Control and Prevention have invited a group of experts to improve and validate this questionnaire.

### 2.4. Sleep Quality

The Chinese-version of the Pittsburgh Sleep Quality Index (PSQI) was used to assess the sleep quality of participants during the past month [26]. The PSQI includes 19 self-evaluation items, which are divided into seven components (sleep quality, sleep latency, sleep duration, sleep efficiency, sleep disorders, use of hypnotic agents and daytime dysfunction) [27]. The total PSQI score is the summed seven component scores with a range of 0–21, the higher the score, the worse the sleep quality is [27]. The sensitivity and specificity of PSQI to distinguish the population with poor sleep quality and the population with good sleep quality were 98.3% and 90.2%, respectively, both with the cut-off points being 7 [26]. Accordingly, we also adopted 7 as a cut-off point in our study, the PSQI score < 7 was defined as good sleep quality, and the PSQI score ≥ 7 was defined as poor sleep quality.

### 2.5. Statistical Analysis

Variables in normal distribution were presented as mean ± standard deviation; variables in non-normal distribution were presented as median (lower quartile-upper quartile), and categorical variables were presented as percentages. Student’s *t*-test and the *Χ*^2^ test were applied to estimate the differences between high and poor sleep quality for the continuous and categorical variables, respectively.

Logistic regression was used to examine the relationship between dietary behaviors and sleep quality, with adjustment for covariates. Multiple linear regression analysis was used to estimate the association of dietary behaviors and the total scores of PSQI with adjustment for age, sex, education level, occupation, marital status, regular exercise, income, BMI, cigarette smoking, hypertension, diabetes, dyslipidemia. Statistical analysis was performed using SPSS version 22.0 (SPSS Inc., Chicago, IL, USA). To avoid false-positive results, *p*-values ≤ 0.005 were considered as statistically significant for multiple testing.

Sleep quality was divided into two categories in logistic regression (using 7 as a cut-off point for PSQI score, PSQI score < 7 was defined as good sleep quality, and the PSQI score ≥ 7 was defined as poor sleep quality [27]), the outcome variable was good sleep quality (poor vs good), and from the OR in the logistic regression, we can directly know that the dietary variable is a risk factor or a protective factor for sleep quality. The PSQI score was treated as a continuous variable in linear regression, with the estimation of the change in PSQI score for every unit change in dietary variables.

## 3. Results

A total of 5160 participants were included in this study. The baseline characteristics of the included participants are presented in Table 1. 8.6% (*n* = 442) of participants reported poor sleep quality. There were statistically significant differences concerning sex, age, education level, occupation, hypertension, and diabetes between participants with good sleep quality and those with poor sleep quality (Table 1).

Participants with poor sleep quality consumed more and alcohol (*p* = 0.001), fewer light vegetables (*p* < 0.001), and less water (*p* = 0.004) than those with higher sleep quality (Table 2).

Table 3 presents the results for the association between dietary behaviors and sleep quality adjusted for age, sex, education level, occupation, marital status, regular exercise, income, cigarette smoking, hypertension, diabetes, and dyslipidemia. Soy sauce intake and alcohol consumption were associated with poor sleep quality (OR = 1.025, 95% CI: 1.008–1.043, *p* = 0.005; OR = 1.448, 95% CI: 1.165–1.800, *p* = 0.001, respectively).

The results of multiple linear regression are presented in Table 4. After adjusting for age, sex, education level, occupation, marital status, regular exercise, income, cigarette smoking, hypertension, diabetes, and dyslipidemia, with every gram increase in each meal consumption of soy sauce, there was a 0.022 point increase in PSQI score (*β* = 0.022, *p* = 0.001). Less intake of dark fruits and water and more intake of alcohol were associated with a higher PSQI score (*β* = −0.394, *p* = 0.001; *β* = −0.246, *p* = 0.001; and *β* = 0.217, *p* = 0.005, respectively).

## 4. Discussion

To the best of our knowledge, this is the first study to investigate the relationship between dietary behaviors and sleep quality in a representative sample of adults in Ningbo. Using a reliable estimation of self-reported sleep quality, 8.6% (*n* = 442) of 5160 participants reported poor sleep quality. Our study found that soy sauce intake and alcohol consumption ≥ 1 day per week were positively associated with poor sleep quality. There was 0.022 point increase in PSQI score for every gram increase in each meal consumption of soy sauce. Fewer intakes of dark fruits and more intake of water and alcohol were associated with the higher PSQI score. The positive association of alcohol consumption with poor quality of sleep in our study is similar to the findings by a prospective cohort study [28]. And the significant association of dark fruits consumption with good sleep quality was also reported in the UK [29]. However, the association between soy sauce intake and sleep quality in Western studies is barely reported, and this may be due to that soy sauce is rarely used in Western foods. The factors screened by the logistic model and linear regression were not completely consistent, due to the different basic principles of the two statistical models. Multiple linear regression models could evaluate the effects of factors on the overall level of PSQI score; whereas the logistic model could estimate the effects of these factors on the risk of developing poor sleep quality.

Those with poor sleep quality were more likely to be female, older, with a lower degree of education, unemployed, with hypertension or diabetes. The proportion of poor sleep quality was lower in our study compared to other studies with 22%–36% of poor sleep quality [30,31]. The lower proportion of poor sleepers in our study may be due to the higher cutoff point to distinguish participants with poor and good sleep quality. Besides, Ningbo is a coastal city, and fish is commonly consumed by local residents [32], so fish consumption could improve sleep quality [15].

Soy sauce intake was positively associated with higher PSQI scores. Tryptophan is an important constituent of soy sauce [33], and an inverse association was observed between tryptophan and PSQI score [34]. Melatonin is considered to regulate the circadian rhythm [35] and is a metabolite of tryptophan, and a decrease in plasma tryptophan level was associated with central fatigue [36]. Therefore, soy sauce intake may affect the melatonin level and affect sleep quality sequentially. After taking too much soy sauce, the concentration of Na+ and Cl- in the blood would rise and accelerate the loss of water, causing more water intake and more trips to the toilet in the middle of the night, which contributes to the poor sleep quality [37]. Compared to Western countries, soy sauce is widely consumed in Asia [38], thus the association between soy sauce and sleep might be very specific for Asia.

Dark fruits consumption was positively associated with the lower PSQI scores. Our findings of the association of dark fruits and sleep quality are similar to the previous studies [39]. Compared to the light fruits, some dark fruits, for example, kiwi, contain a higher proportion of ascorbic acid, which could reduce sleep disorders and improve sleep quality [40]. Tart cherries contain a higher level of exogenous melatonin, which could also improve duration of sleep and reduce disturbance [29]. These mentioned above might be the possible mechanism explaining the effects of dark fruits on improving sleep. However, light fruits were borderline significant in linear regression (*p* = 0.051), we can’t conclude from our findings that there are real differences between light fruits and sleep quality.

Notably, a significantly positive association between PSQI total score and alcohol consumption was also observed in our study. Previous studies also reported significant associations between alcohol consumption and sleep quality [8,9,10,11]. This is due to the fact that alcohol could reduce genioglossal muscle tone, lead to upper airway collapse [41] and increase the upper airway resistance [42], and finally contributed to the risk of obstructive sleep apnoea (OSA) [8]. Additionally, alcohol might influence sleep homeostasis by regulating adenosine (AD) and the wake-promoting basal forebrain (BF), which both are the mediators of sleep homeostasis [9]. However, a recent longitudinal study suggested that the association between sleep duration and alcohol intake might be bidirectional; thus, sleep problems might also lead to alcohol drinking [43]. The association between water intake and PSQI total score was significantly negative in our study, but the explanation remains unclear.

Our results were based on cross-sectional data. In the end, we could not rule out the possibility that people who sleep worse were more prone to eat unhealthy due to changes in appetite, or better sleepers were more healthy in general, and this might reflect in dietary variables. So we could not determine the causal relationship between dietary behaviors and sleep quality. Using the logistic regression, we found that only two dietary variables were significant (soy sauce and alcohol), this might be due to chance.

The following limitations should be acknowledged. Firstly, as is the nature of cross-sectional study, we could not determine the causal relationship. Secondly, other foods, such as milk and meat consumption, which were associated with sleep quality [44,45], however, were not investigated in our study. Thirdly, the PSQI and dietary questionnaire were self-reported rather than measured objectively, so recall bias is a concern. Fourthly, we were unable to control all possible variables, which might influence the relationship between dietary behaviors and sleep quality. Finally, our study did not provide the exact amount of food intake.

To the best of our knowledge, this is the first study to investigate the relationship between dietary behaviors and sleep quality in a representative sample of Chinese adults. PSQI is a reliable estimate of sleep quality with confirmed higher reliability and validity [26]. The participants included in this study were representative of Ningbo; thus, the finding could be generalized to the whole of Ningbo. Furthermore, with adjustment for multivariables, the confounding bias could be diminished.

## 5. Conclusions

In conclusion, 8.5% (*n* = 442) reported poor sleep quality in our study. Intake of soy sauce and alcohol were positively associated with poor sleep quality, and consumption of dark fruits and water were positively associated with good sleep quality. Participants with poor sleep quality should be more careful about the consumption of soy sauce, alcohol, and water. Due to the nature of the cross-sectional study, we cannot determine the causal relationship between dietary behaviors and sleep quality, and future prospective cohort studies are needed to confirm our findings. The maximal limits of the intake of these foods in adults with poor sleep quality should be ascertained by current guidelines.

## Figures and Tables

**Table 1:** The baseline characteristics of the participants between poor and good sleep quality.

Variables	All Participants (*n* = 5160)	Sleep Quality		*p*
		Good Sleep Quality PSQI < 7 (4718)	Poor Sleep Quality PSQI ≥ 7 (442)	
Sex (female)	2821 (54.7)	2516 (53.3)	305 (69.0)	<0.001
Age (years)	46.86 ± 14.96	46.04 ± 14.90	55.53 ± 12.72	<0.001
Above high school	1965 (38.1)	1840 (39.0)	125 (28.3)	<0.001
Unemployed	1104 (21.4)	993 (21.0)	111 (25.1)	<0.001
No spouse	954 (18.5)	868 (18.4)	86 (19.5)	0.170
Regular exercise	2209 (42.8)	2030 (43.0)	179 (40.5)	0.304
Income below population average (%)	3342 (64.8)	3040 (64.4)	302 (68.3)	0.101
BMI	23.66 ± 3.31	23.65 ± 3.31	23.75 ± 3.24	0.540
Smokers	1250 (24.2)	1166 (24.7)	84 (19.0)	0.007
Hypertension	2014 (39.0)	1779 (37.7)	235 (53.2)	<0.001
Diabetes	571 (11.1)	493 (10.4)	78 (17.6)	<0.001
Dyslipidemia	1541(29.9)	1405 (29.8)	136 (30.8)	0.664

PSQI: Pittsburgh Sleep Quality Index; BMI: Body mass index.

**Table 2:** The relationship between dietary behaviors and sleep quality among the participants.

Variables	All Participants (*n* = 5160)	Sleep Quality		*p*
		Good Sleep Quality PSQI < 7 (4718)	Poor Sleep Quality PSQI ≥ 7(442)	
Salt ^b^ (g/meal) M(LQ-UQ)	4.4 (2.8–5.6)	4.4 (2.8–5.6)	4.4 (2.8–6.7)	0.337
MSG ^b^ (g/meal) M (LQ-UQ)	1.1 (0.3–2.2)	1.1 (0.3–2.2)	1.1 (0.3–2.2)	0.710
Edible oil ^b^ (g/meal) M (LQ-UQ)	17.8 (11.1–27.8)	17.7 (11.1–27.8)	17.8 (11.1–27.8)	0.608
Soy sauce ^b^ (g/meal) M (LQ-UQ)	4.4 (2.2–6.7)	4.4 (2.2–6.7)	4.7 (2.2–8.5)	0.015
Dark vegetables ^b^				0.015
<1 time/d	2611 (50.6)	2363 (50.1)	248 (56.1)	
≥1 time/d	2549 (49.4)	2355 (49.9)	194 (43.9)	
Light vegetables ^b^				<0.001
<1 time/d	2328 (45.1)	2052 (43.5)	276 (62.4)	
≥1 time/d	2832 (54.9)	2666 (56.5)	166 (37.6)	
Dark fruits ^b^				0.998
<1 time/d	3934 (76.2)	3597 (76.2)	337 (76.2)	
≥1 time/d	1226 (23.8)	1121 (23.8)	105 (23.8)	
Light fruits ^b^				0.449
<1 time/d	3515 (68.1)	3221 (68.3)	294 (66.5)	
≥1 time/d	1645 (31.9)	1497 (31.7)	148 (33.5)	
Water ^c^				0.004
<4 cups/d	2873 (55.7)	2598 (55.1)	275 (62.2)	
≥4 cups/d	2287 (44.3)	2120 (44.9)	167 (37.1)	
Tea ^c^				0.934
<1 cup/month	918 (17.8)	840 (17.8)	78 (17.6)	
≥1 cup/month	4242 (82.2)	3878 (82.2)	364 (82.4)	
Coffee ^c^				0.239
<1 cup/month	138 (2.7)	130 (2.8)	8 (1.8)	
≥1 cup/month	5022 (97.3)	4588 (97.2)	434 (98.2)	
Alcohol ^c^				0.001
<1 time/month	3867 (74.9)	3565 (75.6)	302 (68.3)	
≥1 time/month	1293 (25.1)	1153 (24.4)	140 (31.7)	
Eating breakfast out ^a^				0.275
Yes	2301 (44.6)	2093 (44.4)	208 (47.1)	
No	2859 (55.4)	2625 (55.6)	234 (52.9)	
Eating lunch out ^a^				0.506
Yes	1482 (28.7)	1349 (28.6)	133 (30.1)	
No	3678 (71.3)	3369 (71.4)	309 (69.9)	
Eating dinner out ^a^				0.532
Yes	913 (17.7)	830 (17.6)	83 (18.8)	
No	4247 (82.3)	3888 (82.4)	359 (81.2)	
Eating midnight snack out ^a^				0.042
Yes	388 (7.5)	344 (7.3)	44 (10.0)	
No	4772 (92.5)	4374 (92.7)	398 (90.0)	

M: Median; LQ: Lower Quartile; UQ: Upper Quartile; ^a^ During the past week; ^b^ During the past month; ^c^ During the past year.

**Table 3:** The relationship between dietary behaviors and poor sleep quality by logistic regression analysis.

Variables	*β*	SE	OR	95% CI	*p*
Salt ^b^	0.003	0.017	1.003	0.969–1.038	0.860
MSG ^b^	0.029	0.036	0.972	0.906–1.042	0.419
Edible oil ^b^	0.001	0.004	1.001	0.993–1.009	0.854
Soy sauce ^b^	0.025	0.009	1.025	1.008–1.043	0.005
Dark vegetables ^b^ (≥1 time/d)	−0.157	0.136	1.170	0.896–1.527	0.249
Light vegetables ^b^ (≥1 time/d)	−0.038	0.137	1.039	0.794–1.360	0.782
Dark fruits ^b^ (≥1 time/d)	−0.074	0.177	0.929	0.656–1.314	0.676
Light fruits ^b^ (≥1 time/d)	0.136	0.159	1.145	0.838–1.564	0.394
Water ^c^ (≥4 cups/d)	−0.149	0.110	0.862	0.695–1.068	0.174
Tea ^c^ (yes)	0.127	0.147	1.135	0.852–1.513	0.386
Coffee ^c^ (yes)	0.254	0.385	1.289	0.606–2.744	0.510
Alcohol ^c^ (≥1 day/wk)	0.370	0.111	1.448	1.165–1.800	0.001
Eating breakfast out ^a^ (yes)	0.143	0.126	1.153	0.901–1.477	0.258
Eating lunch out ^a^ (yes)	−0.298	0.176	0.742	0.526–1.047	0.090
Eating dinner out ^a^ (yes)	−0.009	0.202	0.991	0.666–1.474	0.965
Eating midnight snack out ^a^ (yes)	0.245	0.273	1.278	0.748–2.183	0.370

CI: Confidence Interval; OR: Odds Ratio; ^a^ During the past week; ^b^ During the past month; ^c^ During the past year. Adjusted for age, sex, education level, occupation, marital status, regular exercise, income, cigarette smoking, hypertension, diabetes, dyslipidemia.

**Table 4:** Linear regression of dietary behaviors on PSQI total scores in all participants.

Variables	Unadjusted		Adjusted ^d^	
	*B* ± *SE*	*p*	*B* ± *SE*	*p*
Salt ^b^ (g/meal) M (LQ-UQ)	0.013 ± 0.011	0.248	0.006 ± 0.012	0.588
MSG ^b^ (g/meal) M (LQ-UQ)	0.028 ± 0.024	0.257	−0.040 ± 0.024	0.102
Edible oil ^b^ (g/meal) M (LQ-UQ)	0.008 ± 0.003	0.002	0.004 ± 0.003	0.146
Soy sauce ^b^ (g/meal) M (LQ-UQ)	0.022 ± 0.006	<0.001	0.022 ± 0.006	0.001
Dark vegetables ^b^ (≥1 time/d)	0.077 ± 0.071	0.281	0.086 ± 0.092	0.356
Light vegetables ^b^ (≥1 time/d)	0.121 ± 0.072	0.093	0.005 ± 0.091	0.953
Dark fruits ^b^ (≥1 time/d)	−0.280 ± 0.084	0.001	−0.394 ± 0.119	0.001
Light fruits ^b^ (≥1 time/d)	−0.070 ± 0.077	0.358	−0.213 ± 0.109	0.051
Water ^c^ (≥4 cups/d)	−0.440 ± 0.072	<0.001	−0.246 ± 0.071	0.001
Tea ^c^ (yes)	0.093 ± 0.093	0.320	0.059 ± 0.095	0.535
Coffee ^c^(yes)	−0.331 ± 0.221	0.135	0.294 ± 0.212	0.167
Alcohol ^c^ (≥1 day/wk)	0.244 ± 0.082	0.003	0.217 ± 0.078	0.005
Eating breakfast out ^a^ (yes)	−0.440 ± 0.072	<0.001	0.154 ± 0.081	0.055
Eating lunch out ^a^ (yes)	−0.649 ± 0.079	<0.001	−0.108 ± 0.094	0.252
Eating dinner out ^a^ (yes)	−0.414 ± 0.095	<0.001	0.081 ± 0.110	0.461
Eating midnight snack out ^a^ (yes)	−0.539 ± 0.139	<0.001	0.191 ± 0.142	0.180

^a^ During the past week; ^b^ During the past month; ^c^ During the past year; ^d^ Adjusted for age, sex, education level, occupation, marital status, regular exercise, income, BMI, cigarette smoking, hypertension, diabetes, dyslipidemia.
